# Diffusion constraints in neuroprotection: implications for clinical trials

**DOI:** 10.3389/fphar.2025.1542431

**Published:** 2025-03-24

**Authors:** Daniil P. Aksenov, Evan D. Doubovikov

**Affiliations:** ^1^ Endeavor Health, Evanston, IL, United States; ^2^ Pritzker School of Medicine, University of Chicago, Chicago, IL, United States; ^3^ Northwestern University, Evanston, IL, United States

**Keywords:** edaravone, diffusion, stroke, ischemic, infarction, hypoxia, neuroprotection

The recent editorial in JAMA Neurology “Promising Efforts to Define a Novel Approach to Neuroprotection for Acute Ischemic Stroke” (Anderson and Song, 2024) reviewed the effects of edaravone and expressed cautious hope for its neuroprotective potential in acute ischemic stroke. While the findings from the clinical trials (Fu et al., 2024) and preclinical studies are promising, we would like to raise critical questions regarding edaravone’s efficacy as a neuroprotective agent in humans and the broader applicability of other neuroprotectants. These results are compelling, yet we believe they warrant closer examination, particularly in the context of drug diffusion and perfusion.

Ischemic stroke initiates a rapid sequence of events leading to brain injury. Within minutes of arterial blockage, the ischemic core experiences severe oxygen and glucose deprivation, resulting in neuronal death. Surrounding this core is the ischemic penumbra, a region with reduced perfusion that remains metabolically active but functionally compromised. Over hours to days, without intervention, the penumbra may succumb to irreversible damage due to ongoing energy failure, excitotoxicity, oxidative stress, and inflammation. This progression underscores the critical importance of timely therapeutic interventions to salvage penumbral tissue and improve clinical outcomes.

The effectiveness of edaravone, like many neuroprotective agents, relies heavily on its ability to preserve tissue with reduced perfusion, reduce edema, and even partially treat ischemic regions. Indeed, profound effects have been demonstrated in preclinical models, where edaravone significantly reduced infarction size in rat brains (Wu et al., 2000). However, such findings should not obscure the significant translational challenges posed by scaling these results to humans.

Despite extensive efforts to develop neuroprotective therapies for hypoxia-related brain damage, the incidence of hypoxic brain events remains high, and the outcomes of neuroprotective clinical trials continue to disappoint. Neuroprotective compounds often show success in animal models, yet consistently fail to deliver the same results in human clinical trials for conditions like ischemic stroke. This discrepancy has puzzled researchers for decades, leading to repetitive trials without fully addressing the reasons behind these failures (Anderson and Song, 2024).

Historically, researchers have explained this translational gap through differences in study design, such as variations in dosage, timing, or patient selection (Drummond et al., 2000; Gorelick, 2000). While these are valid considerations, they overlook a critical issue: local pharmacokinetics of neuroprotective drugs within brain tissue. Drug diffusion distances in brain tissue, in addition to crossing the blood-brain barrier (BBB), play a pivotal role in the failure of neuroprotective strategies in humans. Only recently have findings begun to reveal the full impact of these diffusion constraints, particularly in the context of brain size and the scaling of diffusion distances across species (Wu et al., 2023; Doubovikov and Aksenov, 2024).

In the absence of functional vascular delivery, neuroprotective drugs that cross the BBB must rely on passive diffusion from healthy tissue to reach ischemic areas. The distance a drug can diffuse depends on factors such as its molecular weight, universal brain tissue properties, and the level of perfusion. Importantly, these diffusion distances are not adjustable and remain constant across species due to the fundamental properties of molecular movement and the inherent structure of brain tissue, such as tissue density and the extracellular matrix. Despite the vast differences in brain size between species, diffusion distances governed by Fick’s law of diffusion do not scale proportionally. The assumption that drug efficacy can be scaled from rodents to humans without addressing these constraints has contributed to confusion in neuroprotective trials.

For smaller brains, such as those in commonly used preclinical models, diffusion distances may cover a considerable portion of the area with reduced perfusion, leading to effective neuroprotection. In contrast, in the human brain, with its significantly larger volume—exceeding 1,260 cubic centimeters—the same diffusion distance may cover only a fraction of the penumbra or ischemic core. If a drug diffuses only a few millimeters, it means that a drug capable of covering 10%–30% of the affected area in a smaller preclinical model may only reach less than 0.1% of the affected area in the much larger human brain. This stark contrast underscores the significant challenge posed by diffusion limitations, which create a major barrier to effectively translating neuroprotective therapies from smaller animal models to human clinical applications. Although intermediate species such as primates [e.g., male cynomolgus monkeys with brain volumes around 150 cubic centimeters (Sakane et al., 2020; Yoshikawa et al., 2005)] have larger brains than rodents, their brain sizes remain substantially smaller than those of humans. Consequently, even in primates, while the limited diffusion distance may allow for effective or partially effective treatment, it still underscores a significant barrier to translating neuroprotective therapies from animal models to clinical practice.

Edaravone is considered to have a low molecular weight, which theoretically facilitates its diffusion. However, based on recent findings, even oxygen—under optimal experimental conditions, including reduced neuronal consumption from general anesthesia and 100% inspired oxygen—can diffuse only a few millimeters (Doubovikov and Aksenov, 2024). Notably, oxygen has a molecular weight of 32 g/mol, which is significantly lower than that of edaravone (MW = 174 g/mol). Given this disparity, we question if edaravone can diffuse far enough to provide meaningful neuroprotection in human brains. To our knowledge, there is no published data on the diffusion properties of edaravone in brain tissue, which is surprising because this factor should be considered by any clinical trials before they start. If edaravone can diffuse only for a couple of millimeters, this limitation would render it effective only in regions with preserved vascularization, leaving most of the hypoxic area in humans untreated. Moreover, it is important to note that molecular weight is not the sole determinant of tissue penetration; factors such as lipophilicity, charge, protein binding, and metabolism also critically influence a drug’s ability to permeate brain tissue. However, in the absence of a diffusion model that integrates comprehensive data on these additional parameters for edaravone—or direct measurements of its actual diffusion in brain tissue *in vivo*—the extent of its effective distribution remains uncertain.

It is also important to consider the role of boundary effects and partial perfusion in modulating drug efficacy. In cases where the ischemic lesion is small or consists of multiple microinfarcts, the higher boundary-to-volume ratio enables the drug to cover a larger fraction of the affected tissue. Even a limited diffusion distance may suffice to reach most of the lesion’s periphery, where residual perfusion persists. Furthermore, the combined influence of active blood flow and passive diffusion in these boundary zones can enhance local drug concentrations, thereby promoting neuroprotection. Although this mechanism might account for the observed effects of edaravone, it is unlikely to be as effective as seen in small animal studies unless edaravone’s diffusion distance in human tissue is significantly greater than the few millimeters.

This observation has significant implications for future clinical research. Strategies to improve drug delivery—perhaps through localized delivery systems, enhanced diffusion mechanisms, or novel approaches like nanoparticles—are urgently needed. Until these physical limitations are addressed, pharmacological neuroprotective strategies are unlikely to fulfill their potential in clinical trials. Specifically, for edaravone, we recommend further studies to clarify its pharmacokinetics, particularly in regions with limited or absent vascular supply. Moreover, for all potential neuroprotective compounds, comparative studies with varying infarction sizes and perfusion levels could also provide valuable insights into its translational potential. Without this critical understanding, clinical trials risk failure due to overlooked physical barriers, as has been demonstrated repeatedly in this field.
